# B-1 Cell Immunoglobulin Directed Against Oxidation-Specific Epitopes

**DOI:** 10.3389/fimmu.2012.00415

**Published:** 2013-01-09

**Authors:** Dimitrios Tsiantoulas, Sabrina Gruber, Christoph J. Binder

**Affiliations:** ^1^CeMM Research Center for Molecular Medicine of the Austrian Academy of SciencesVienna, Austria; ^2^Department of Laboratory Medicine, Medical University of ViennaVienna, Austria

**Keywords:** B-1 cells, oxidation-specific epitopes, natural IgM antibodies, malondialdehyde, phosphocholine, oxidized low-density lipoprotein, apoptotic cells

## Abstract

Natural antibodies (NAbs) are pre-existing antibodies with germline origin that arise in the absence of previous exposure to foreign antigens. NAbs are produced by B-1 lymphocytes and are primarily of the IgM isotype. There is accumulating evidence that – in addition to their role in antimicrobial host defense – NAbs exhibit important housekeeping functions by facilitating the non-immunogenic clearance of apoptotic cells as well as the removal of (neo-)self antigens. These properties are largely mediated by the ability of NAbs to recognize highly conserved and endogenously generated structures, which are exemplified by so-called oxidation-specific epitopes (OSEs) that are products of lipid peroxidation. The generation of OSEs as well as their interaction with the immune system have been studied extensively in the context of atherosclerosis, a chronic inflammatory disease of the vascular wall that is characterized by the accumulation of cellular debris and oxidized low-density lipoproteins (OxLDL). Both apoptotic cells as well as OxLDL carry OSEs that are targeted by NAbs. Therefore, OSEs represent stress-induced neo self-structures that mediate recognition of metabolic waste (e.g., cellular debris) by NAbs, allowing its safe disposal, which has fundamental implications in health and disease.

## B-1 Cells are the Producers of Natural IgM Antibodies

In contrast to conventional B-2 cells, B-1 cells have a self-replenishing capacity, are localized to the pleural and peritoneal cavities, and exhibit different activation requirements (Martin and Kearney, 2001; Montecino-Rodriguez and Dorshkind, 2006). A major function of B-1 cells is their capacity to secrete natural antibodies (NAbs), which are produced very early in life. NAbs are pre-existing antibodies, whose variable regions are encoded by germline (or near germline) encoded variable genes. They are termed “natural” due to the fact that their development does not depend on foreign antigen exposure, and therefore a normal NAb repertoire is found at equivalent titers in the blood of mice housed in completely “germ”-free conditions (Avrameas, 1991; Notkins, 2004). NAbs are primarily of the IgM isotype and bind both self and foreign antigens by recognizing highly conserved structures of nucleic acids, (glyco)proteins, and (phospho)lipids that are present on the surface of microbes such as bacteria, viruses, and fungi (Briles et al., 1982; Choi and Baumgarth, 2008; Rapaka et al., 2010), and endogenous (neo-)self antigens, respectively. Through this, NAbs are critically involved in the first line defense against microbial pathogens and exhibit “housekeeping” functions by regulating tissue homeostasis (Avrameas, 1991; Notkins, 2004).

For example, mice deficient in secreted IgM (sIgM^-/-^) show increased susceptibility to acute peritonitis in a cecal ligation puncture model (Jenck et al., 1998), but also a stronger tendency of developing an auto-immune phenotype upon LPS injection or when crossed onto the lupus prone lpr background (Boes et al., 2000). Moreover, sIgM^-/-^ mice display impaired T-cell responses when immunized with suboptimal dosages of antigen and an abnormal B cell development with increased B-1 and marginal zone B cell numbers. These data also point to an important role in immune regulation. Due to their specificity for altered self-structures, NAbs have also been suggested to play a role in tumor immunosurveillance (Vollmers and Brandlein, 2007; Schwartz-Albiez et al., 2008) as well as in neurodegenerative disorders such as Alzheimer’s disease (Szabo et al., 2008), which are conditions that are characterized by the alteration of self-structures.

## Oxidation-Specific Epitopes

In the last years it has become increasingly apparent that lipid peroxidation results in the generation of specific structures that are recognized by pattern recognition receptors (PRR) of the innate immune system (Chou et al., 2008; Weismann and Binder, 2012). These include humoral responses, such as NAbs, complement factor H, C reactive protein, as well as cellular receptors, such as scavenger receptor CD36 and TLR-4. Thus, oxidation-specific epitopes (OSEs) constitute a novel class of damage-associated molecular patterns (DAMPs) targeted by both PRRs as well as soluble pattern recognition proteins (Miller et al., 2011). The peroxidation of phospholipids occurs ubiquitously as a physiological process, but is strongly enhanced during pathological situations. Thus, oxidation products are generated and accumulate in essentially all inflammatory settings, including atherosclerosis, pulmonary, renal, and liver diseases, and diseases affecting the central nervous system, such as multiple sclerosis and Alzheimer’s disease (Neale et al., 1994; Palinski et al., 1996; Dei et al., 2002; Casado et al., 2006; Sanchez et al., 2006; Imai et al., 2008; Haider et al., 2011). As a consequence of the inflammatory response, highly reactive lipid peroxidation products are generated, which in turn are capable of modifying autologous proteins and lipids leading to the generation of altered self-structures. These processes were initially studied and characterized in oxidized low-density lipoproteins (OxLDL), which is a prominent component of atherosclerotic lesions. During atherogenesis, LDL accumulates in the vascular wall, where it is subjected to oxidative modifications leading to the generation of OxLDL. The oxidation of LDL has been shown to result in the formation of numerous lipid peroxidation-derived structures (OSEs) that are capable of triggering robust immune responses (Chou et al., 2009). For example, when the oxidation-prone sn-2 polyunsaturated fatty acid of phosphatidylcholine (the main phospholipid of LDL) undergoes oxidation, breakdown products, such as malondialdehyde (MDA) with its many advanced condensation products, 4-hydroxynonenal (4-HNE), and the remaining “core aldehyde” 1-palmitoyl-2-(5-oxovaleroyl)-sn-glycero-3-phosphocholine (POVPC) are formed (Horkko et al., 2000). These then modify autologous molecules and generate (often repetitive) adducts that are recognized by various immune receptors in a hapten-specific manner. In order to study the antigenicity of OxLDL, a variety of model antigens have been established, including the frequently used CuSO_4_-oxidized LDL (CuOx-LDL) and malondialdehyde-modified LDL (MDA-LDL). Other model antigens include bovine serum albumin modified with either MDA, 4-HNE, or POVPC.

Importantly, cells undergoing apoptotic cell death are exposed to enhanced oxidative stress as well, which also leads to the generation of oxidized lipids, such as cardiolipin, phosphatidylserine, and phosphatidylcholine (Chang et al., 2004; Kagan et al., 2004). Indeed, all of the OSEs described above have also been found on the membranes of apoptotic cells and cellular debris, where they represent tags by which components of innate immunity can distinguish dying cells from viable cells – a property that is critically important for immune homeostasis.

## NAbs and OSEs

The first identification of NAbs with specificity for OSEs came from the seminal observation that atherosclerotic Apolipoprotein E deficient mice (Apoe^-/-^) were found to have very high titers of autoantibodies to epitopes of OxLDL (Palinski et al., 1996). This enabled Dr. Witztum’s laboratory to clone a large set of hybridomas producing IgM antibodies with specificity for OxLDL model antigens from the spleens of non-immunized atherosclerotic Apoe^-/-^ mice. Of these hybridomas, a CuOx-LDL-specific clone, termed E06, was found to specifically bind the phosphocholine (PC) head-group of oxidized but not native unoxidized phosphatidylcholine, indicating that E06 binds to OxLDL via oxidized phospholipids (Friedman et al., 2002). A detailed sequence analysis of the CDR3 region of E06 revealed it to be of 100% germline origin and identical to a prototypic NAb called T15 (Shaw et al., 2000). T15 (an IgA) is a well known PC-specific NAb that is exclusively derived from B-1 cells (Masmoudi et al., 1990). It has been shown to bind *S. pneumoniae*, which contain PC as prominent constituent of the (lipo)teichoic acid components of the cell wall polysaccharide, thereby protecting mice from pneumococcal infections (Briles et al., 1982). Moreover, immunization of cholesterol-fed atherosclerosis prone LDL receptor deficient (Ldlr^-/-^) mice with heat-killed extracts of R36a *S. pneumoniae* containing PC resulted in high anti-OxLDL IgM titers, which were found to be nearly all of the T15/E06 clonotype, and significantly reduced atherosclerotic plaque development (Binder et al., 2003). This atheroprotective effect of T15/E06 Abs was further confirmed by a study in which passive infusion of a monoclonal T15/E06id^+^ IgM preparation reduced vein graft atherosclerosis in Apoe^-/-^ mice (Faria-Neto et al., 2006). Several mechanisms have been proposed by which T15/E06 mediates this protection from atherosclerosis. Because PC is also found on apoptotic cell membranes, T15/E06 IgM may inhibit atherogenesis by facilitating a proper clearance of apoptotic cells (Shaw et al., 2000; Ogden et al., 2005; Chen et al., 2009b). Indeed, impaired clearance of apoptotic cells has been found to promote atherosclerotic lesion formation (Ait-Oufella et al., 2007; Thorp et al., 2008). In line with this, it has been shown that T15/E06 IgM Abs enhance the clearance of apoptotic cells by a mechanism that is dependent on the co-recruitment of C1q and mannose-binding lectin (Chen et al., 2009b). Moreover, it has been shown that T15/E06 is able to restore the complement dependent clearance of apoptotic cells in B cell-deficient and sIgM^-/-^ mice, respectively (Ogden et al., 2005; Chen et al., 2009a). Mice deficient in sIgM when crossed onto the Ldlr^-/-^ background develop accelerated atherosclerosis, further supporting the protective role of NAbs in hypercholesterolemia-induced oxidative stress (Lewis et al., 2009). In addition, splenectomized Ldlr^-/-^ mice, which have strongly reduced peritoneal B-1a cell numbers and consequently reduced serum IgM levels, develop accelerated atherosclerosis that could be rescued by adoptive transfer of B-1a cells isolated from naïve donors but not from sIgM^-/-^ mice. It has been proposed that a more efficient clearance of cellular debris in mice reconstituted with wild type B-1a cells (which include OSE-specific B-1 cells) is responsible for the protective effect (Kyaw et al., 2011).

Apart from these clearance properties, T15/E06 has also been demonstrated to exhibit protective functions by neutralizing the pro-inflammatory effects of oxidized phospholipids. For example, macrophages respond to oxidized 1-palmitoyl-2-arachidonoyl-*sn*-glycero-3-phosphorylcholine (OxPAPC) stimulation by secreting IL-6, and this pro-inflammatory effect of OxPAPC was inhibited upon co-treatment with T15/E06 Abs (Imai et al., 2008). These protective properties of T15/E06 involve its ability to block the recognition of OxPAPC by the macrophage scavenger receptor CD36 (Horkko et al., 1999; Binder et al., 2003; Stewart et al., 2010). Moreover, apoptotic cells or blebs (also called microvesicles) that carry OSEs were shown to activate endothelial cells resulting in increased monocyte adhesion (Huber et al., 2002; Chang et al., 2004; Liu et al., 2012), and T15/E06 has been shown to inhibit this effect (Huber et al., 2002; Chang et al., 2004).

Additional NAbs with specificity for other OSEs have also been identified. A monoclonal IgM NAb named E014 cloned from the spleens of atherosclerotic Apoe^-/-^ mice has been shown to bind MDA-LDL as well as apoptotic cells (Palinski et al., 1996; Chang et al., 1999). Moreover, E014 can bind to apoptotic blebs and – as recently demonstrated – to cholesterol induced microvesicles that carry MDA-epitopes on their surface (Huber et al., 2002; Liu et al., 2012). Both may represent important pro-inflammatory targets of NAbs in atherosclerotic lesions. Finally, another germline-encoded NAb with specificity for OxLDL has been isolated from the spleens of atherosclerotic Ldlr^-/-^ mice. This clone, named LR01, was found to be directed against oxidized but not native cardiolipin, and was also shown to bind apoptotic but not viable cells (Tuominen et al., 2006).

All these NAbs have remarkable specificity for their cognate antigens, as they do not exhibit cross-reactivity. For example, E06 binds PC-adducts but not MDA-adducts, while E014 binds MDA-adducts but not PC-adducts. Although these OSEs are often present on the surface of the same antigens (e.g., apoptotic cells, OxLDL), the selectivity for different but ubiquitous oxidation-specific structures underscores the critical need of NAbs recognizing OSEs (Binder, 2010; see Table 1).

**Table 1:** **Natural IgM antibodies recognize oxidation-specific epitopes present on apoptotic cells and OxLDL and modulate their inflammatory properties**.

NAbs	OSEs	Properties/Functions
T15/E06	PC head-group of oxidized phosphatidylcholine	Blockage of OxLDL uptake by macrophages (Horkko et al., 1999; Binder et al., 2003) Binding to OxLDL and apoptotic cells and atherosclerotic lesions (Palinski et al., 1996; Chang et al., 1999, 2004) Inhibition of apoptotic cell/blebs mediated endothelial cell activation resulting in decreased monocyte adhesion (Huber et al., 2002; Chang et al., 2004) Inhibition OxPAPC-induced IL-6 secretion by lung tissue macrophage (Imai et al., 2008) Enhancement of *in vivo* apoptotic cell clearance in B cell-deficient (μMT) and sIgM^-/-^ mice (Ogden et al., 2005; Chen et al., 2009a) Protection from atherosclerosis (Binder et al., 2003; Faria-Neto et al., 2006) Clinical score reduction in collagen induced arthritis (Chen et al., 2009a)
E014	Malondialdehyde	Binding to OxLDL, apoptotic cells, and atherosclerotic lesions (Palinski et al., 1996) Binding to apoptotic and cholesterol induced microvesicles (Huber et al., 2002; Liu et al., 2012)
NA-17	Malondialdehyde	Binding to OxLDL and apoptotic cells (Chou et al., 2009) Enhancement of clearance of apoptotic cells by macrophages in Rag1^-/-^ mice *in vivo* (Chou et al., 2009)
LR01	Oxidized cardiolipin	Binding to OxLDL, apoptotic cells, and atherosclerotic lesions (Tuominen et al., 2006)

To directly address the relative contribution of OSE-specific NAbs to the entire pool of B-1 cell derived NAbs, we selectively reconstituted Rag1^-/-^ mice with B-1 cells from naïve donor mice. Detailed analysis of the serum of reconstituted mice solely expressing B-1 cell derived Ig, revealed that ∼30% of all IgM Abs had specificity for different OSEs, including MDA, 4-HNE, and PC. Notably, MDA-type adducts were found to be the most prominently bound epitopes among the tested antigens. These results were also supported by the finding that nearly 12% of all IgM secreting cells in the spleens of reconstituted mice had specificity for MDA-epitopes, and similar results were also observed with splenocytes of naïve unchallenged mice. Furthermore, an MDA-specific NAb, termed NA-17, was cloned from the spleens of B-1 cell reconstituted mice, and CDR3 sequence characterization revealed complete germline origin of the V-D-J rearrangement of the heavy chain and only one nucleotide insertion (C) at the splice site of the V_L_ and J_L_ germline gene segments of the light chain. This NAb was also shown to bind apoptotic cells and to promote their clearance by peritoneal macrophages in Rag1^-/-^ mice *in vivo* (Chou et al., 2009).

**Figure 1:**
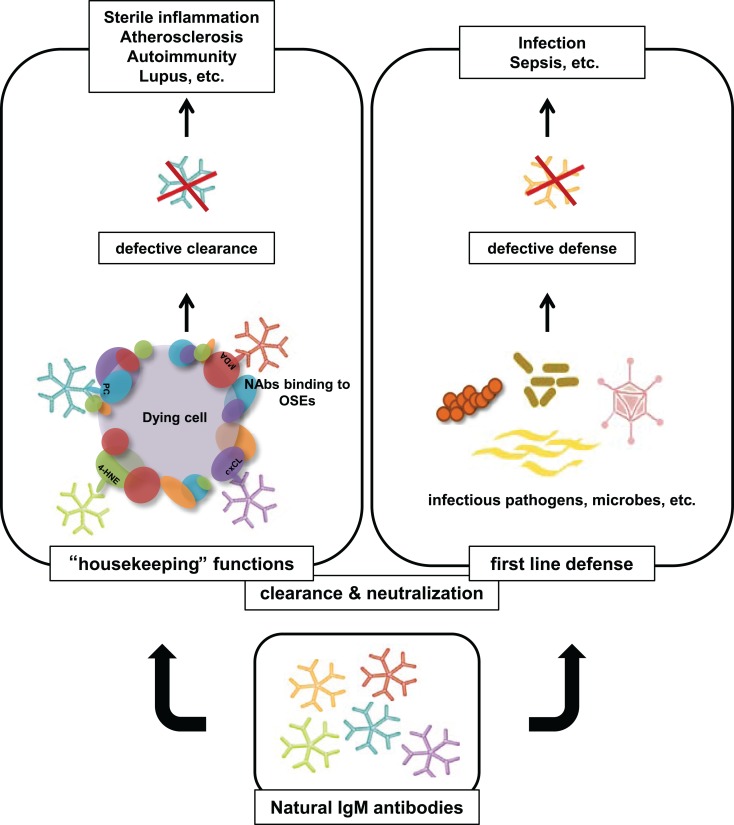
**Overview of protective properties of NAbs specific to OSEs**. NAbs exhibit housekeeping and host defense functions by targeting OSEs on the surface of apoptotic cells, self antigens, and microbial agents. Insufficient NAbs titers may lead to impaired clearance of cellular debris and reduced protective capacity from invading pathogens. Accumulation of apoptotic material can trigger auto-immune responses and promote chronic inflammatory diseases.

In summary, a large part of NAbs has been shown to recognize different OSEs that are present on OxLDL, apoptotic cells, and cellular debris. Similar to PC epitopes on *S. pneumoniae*, equivalent structures for other OSEs such as MDA have been suggested to be present on the surface of *P. gingivalis* (Turunen et al., 2012). Therefore, through the recognition of the same epitopes, NAbs have the capacity to protect from the pathological accumulation of self antigens and defend against microbial infections (see Figure 1). An impaired function of NAbs may therefore result in chronic inflammation as well as increased susceptibility to certain infections.

## B-1 Cells and OSE-Specific NAbs in Human Health and Disease

Circulating antibodies with specificity for OSEs, such as PC and MDA have been shown to be also present in healthy subjects. We have previously shown that a major proportion (∼50%) of IgM in human umbilical cord blood, which serves as a good surrogate for NAbs in humans, binds to apoptotic cells by their ability to recognize MDA-adducts that are present on their surface. Interestingly, we also found that compared to adult blood NAbs in umbilical cord blood are particularly enriched for specificities to CuOx-LDL and MDA-LDL, which are prototypic B-1 cell antigens in mice (Chou et al., 2009). Recently, the human B-1 cell equivalent has been identified. Griffin et al. analyzed purified human B cell populations for B-1 cell characteristics that have been described in mice, including spontaneous IgM secretion, efficient T-cell stimulation, and tonic intracellular signaling. Based on these they determined a small population of B cells, present in umbilical cord and adult peripheral blood, and identified them as CD20^+^CD27^+^CD43^+^CD70^−^ (Griffin et al., 2011). Of note, these cells display B cell receptors (BCRs) with binding specificities for a dsDNA-mimotope and for the classic B-1 cell antigen PC, which is the cognate antigen of the murine T15/E06 NAb. These data suggest that OSE-specific IgM are also secreted by B-1 cells in humans.

Accumulating evidence points to a protective role for OSE-specific IgM antibodies in cardiovascular disease. CuOx-LDL- and MDA-LDL-specific IgM, but not IgG, titers in the serum of patients were shown to be inversely correlated with carotid intima-media thickness and the risk of developing a >50% diameter stenosis in the coronary arteries, respectively (Karvonen et al., 2003; Tsimikas et al., 2007). Moreover, anti-PC IgM antibody titers have been shown to be inversely correlated with cardiovascular disease risk in lupus patients (Anania et al., 2010; Gronwall et al., 2012) as well as with an increased incidence of strokes (Fiskesund et al., 2010) and heart attacks (Gronlund et al., 2009). These epidemiological findings are particularly interesting, as human B-1 cells that produce NAbs have been found to decline with age (Griffin et al., 2011). Thus, it can be speculated that in older individuals the capacity to produce protective NAbs is reduced, which could further enhance the cardiovascular risk.

## Conclusion

B-1 cell derived NAbs exhibit crucial housekeeping functions by recognizing OSEs that are present on apoptotic cells and OxLDL. As a result of this binding capacity, NAbs facilitate the non-inflammatory clearance of apoptotic material preventing the accumulation of debris that may lead to auto-immune/inflammatory responses. The clearance properties of NAbs are particularly important in the context of chronic inflammation, which is characterized by increased oxidative stress. In such conditions the increased generation of neo-self antigens as well as the accumulation of debris following tissue damage are the main sources of inflammation. Moreover, the clearance capacity of NAbs may not be sufficient in advanced chronic inflammatory conditions. Therefore the identification of mechanisms and methods that enhance NAb production may provide a novel approach for the treatment of such pathological conditions.

## Conflict of Interest Statement

The authors declare that the research was conducted in the absence of any commercial or financial relationships that could be construed as a potential conflict of interest.
